# Evaluation of Chemotherapy Response with Serum Squamous Cell Carcinoma Antigen Level in Cervical Cancer Patients: A Prospective Cohort Study

**DOI:** 10.1371/journal.pone.0054969

**Published:** 2013-01-22

**Authors:** Mingzhu Yin, Yan Hou, Tao Zhang, Changyi Cui, Xiaohua Zhou, Fengyu Sun, Huiyan Li, Xia Li, Jian Zheng, Xiuwei Chen, Cong Li, Xiaoming Ning, Kang Li, Ge Lou

**Affiliations:** 1 Department of Gynecology and Oncology, The Affiliated Tumor Hospital of Harbin Medical University, Harbin, Heilongjiang Province, China; 2 Department of Epidemiology and Biostatistics, Harbin Medical University, Harbin, Heilongjiang Province, China; 3 Laboratory of Genetics, National Institute on Aging, National Institutes of Health, Baltimore, Maryland, United States of America; 4 Department of Biostatistics, University of Washington, Seattle, Washington, United States of America; 5 Department of Radiotherapy Oncology, The Affiliated Tumor Hospital of Harbin Medical University, Harbin, China; 6 Department of Cardiology, The Second Affiliated Hospital of Harbin Medical University, Harbin, China; 7 Department of Pathology, The Affiliated Tumor Hospital of Harbin Medical University, Harbin, China; IPO, Inst Port Oncology, Portugal

## Abstract

MRI does not always reflect tumor response after chemotherapy. Therefore, it is necessary to explore additional parameters to more accurately evaluate tumor response for the subsequent clinical determination about radiotherapy or radical surgery. A training cohort and an external validation cohort were used to examine the predictive performance of SCC-ag to evaluate tumor response from teaching hospital of Harbin Medical University. The study included 397 women with SCC (age: 28–73 years). Patients consecutively enrolled between August 2008 and January 2010 (n = 205) were used as training cohort. Patients consecutively enrolled between February 2010 and May 2011 (n = 192) were used as validation cohort. A multivariate regression analysis of the data from the training cohort indicated that serum SCC-ag level is an independent factor for neo-adjuvant chemotherapy (NACT) response. Analysis of the data from the validation cohort suggested that chemotherapy response could be more accurately predicted by SCC-ag than by magnetic resonance imaging (MRI) (sensitivity (Se): 0.944 vs. 0.794; specificity (Sp): 0.727 vs. 0.636; positive predictive value (PPV): 0.869 vs. 0.806; negative predictive value (NPV): 0.873 vs. 0.618; the area under ROC curve (AUC): 0.898 vs. 0.734). Combining SCC-ag with MRI was more powerful than MRI alone (Se: 0.952 vs. 0.794; Sp: 0.833 vs. 0.636; PPV: 0.916 vs. 0.806; NPV: 0.902 vs. 0.618; AUC: 0.950 vs. 0.734). Our study indicates that serum SCC-ag level is a sensitive and reliable measure to evaluate cervical cancer response to chemotherapy. Using SCC-ag in combination with MRI findings further improves the predictive power.

## Introduction

Neoadjuvant chemotherapy (NACT) could create chances for curative resection of initially non-resectable tumors [1]–[4]. However, approximately 30% of the patients with squamous cervical cancer (SCC) are non-responsive to chemotherapy [5], [6]. For patients not responding to neoadjuvant chemotherapy, attempt to remove the tumor with surgery could be disastrous.

MRI is the golden standard to evaluate tumor response to chemotherapy. For cervical cancer patients receiving neoadjuvant chemotherapy, MRI findings are used to determine eligibility of the patients for subsequent resection [7]–[9]. MRI is prone to false-positive results, i.e tumor appears to be decreased in size upon MRI imaging, but actually did not change or even have increased in size based on post-surgical pathological examination, or false-negative results, e.g, in patients with “no residual disease” as judged by MRI imaging, histologic examination detected lesions that measured >1 cm in 8% of the case [10]. Integrated ^18^F-fluorodeoxyglucose positron emission tomography/computed tomography (FDG-PET/CT) imaging improves the evaluation accuracy of tumor volume after chemotherapy [11]. The expense, however, has limited its use, especially in developing countries.

The squamous cell carcinoma antigen (SCC-ag), which serves as a serological marker for squamous cell cervical cancer, is a sub-fraction of the tumor antigen TA-4, which is a 48 kDa glycoprotein that was first isolated by Kato and Torigoe [12]. This antigen is reported to be closely related to clinical staging or the tumor spread as well as the tumor response of advanced squamous disease to radiation or chemotherapy [13]–[15] and can be used to predict the survival and tumor recurrence during the follow-up period [16]–[20].

In the current study, we examined the sensitivity and reliability of using serum SCC-ag level to evaluate response to chemotherapy in patients with cervical cancer. The study included a training cohort of 205 subjects and an external validation cohort of 192 subjects. A random forest model was used to test the hypothesis that SCC-ag level in combination with MRI improves the evaluation of response to chemotherapy.

## Materials and Methods

### Inclusion Criteria

Patients were enrolled in this study if they satisfied all following inclusion criteria: 1) a diagnosis of stage IB2-IIB SCC (FIGO classification); 2) no prior hysterectomy, pelvic radiotherapy, systemic chemotherapy or medical contraindications to chemotherapy. All patients have signed up the written informed consent. The study was approved by the Institutional Review Board. All patients received NACT treatment following radical dissection, and underwent MRI and SCC-ag examinations. NACT regimen consisted of three cycles of paclitaxel and carboplatin treatment. On the first day of each cycle, patients received paclitaxel at 150 mg/m^2^ intravenously (IV) over a period of 3 hours plus carboplatin (area under the serum concentration-time curve: 5) over a period of 30 minutes. Blood pressure, ECG and blood oxygen saturation were monitored during the infusion. Cycles were separated by 3 weeks.

### Training and Validation Cohorts

The training cohort includes all patients with a diagnosis of stage IB2-IIB SCC between August 2008 and January 2010. The validation cohort included all patients diagnosed with stage IB2-IIB SCC between February 2010 and May 2011.

### Magnetic Resonance Imaging

All patients underwent MRI scans at the initial visit using a 1.5-T NVi/CVi magnetic resonance machine (GE, Waukesha, Wisconsin, USA) with a standard phased array torso coil. An additional MRI scan was carried out upon completion of NACT. Briefly, a sagittal T1- and T2-weighted fast spin echo acquisition was obtained with a 24×24 cm field of view (FOV), an echo time (TE) of 97.6 cm, and a repetition time (TR) of 1600 ms. Slices (4 mm) were acquired with 1-mm gap using a 256×256 matrix. The scan ranged from the iliac crests to the pubic symphysis. Based on the sagittal view, axial slices were obtained through the tumor using a T2-weighted fast recovery fast spin echo sequence with an FOV of 32×32 cm, a TE/TR of 83.6/4520 ms, 3-mm slices (no gap) and a 256×256 matrix.

Once the tumor was fully visualized, diffusion-weighted images (DWIs) were acquired in straight axial and sagittal planes (planned on the T2-weighted sagittal scan) and centered through the middle of the tumor using an epi-based diffusion tensor imaging sequence. The epi-based sequence was limited to straight axial slices. All slices were acquired with a 40×40-cm FOV, a 2-mm slice thickness, a TR/TE of 6800 ms/70 ms, and a 160×256 matrix. Data were acquired with a *b* value of 1000. The ADC value was obtained from diffusion tensor images on each slice.

### Clinical Response Evaluation and Imaging Analysis

**Figure 1:**
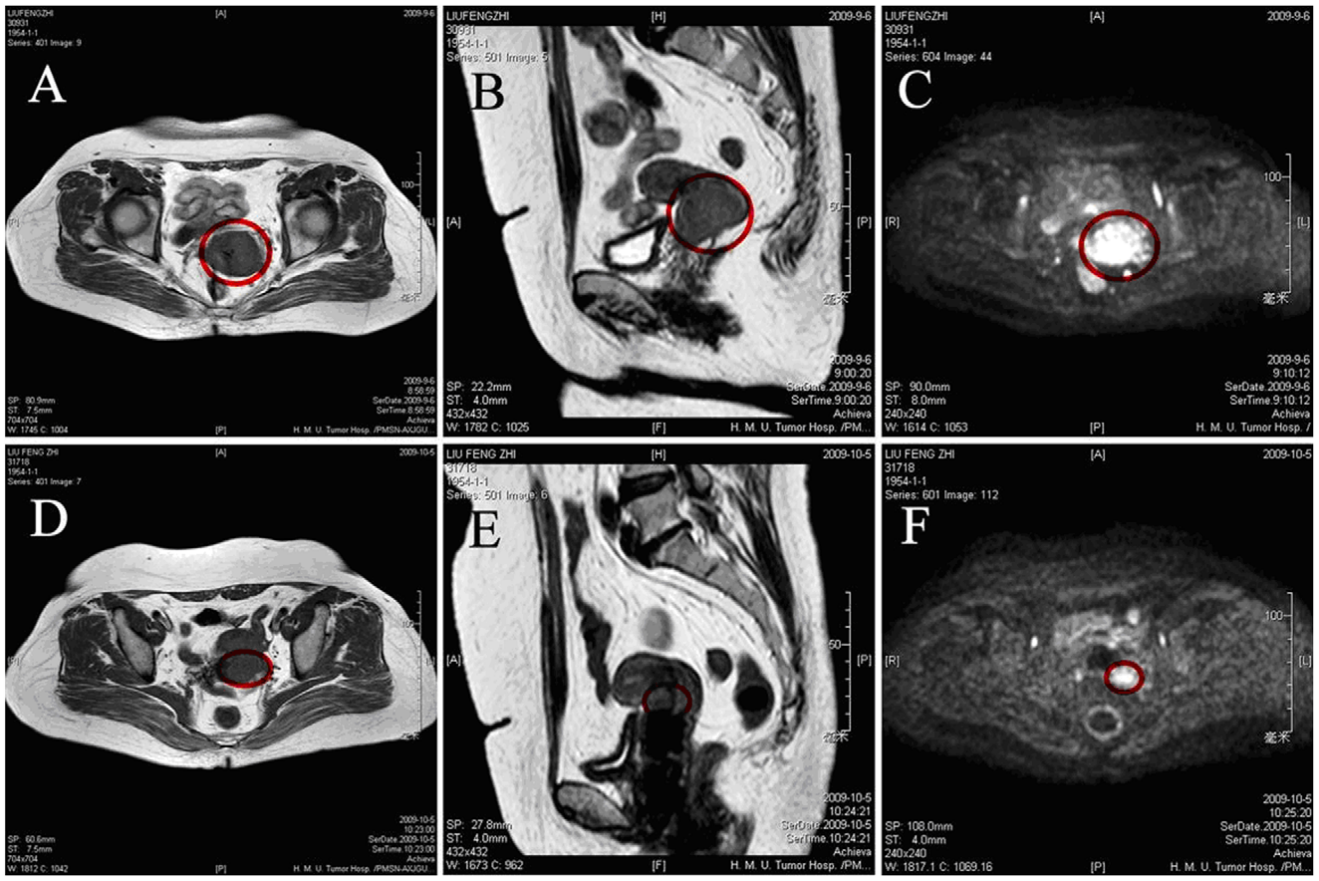
Conventional and DW-MRI of the same lesion from a 55-year-old woman undergoing NACT. (A)–(C): pretreatment axial (A) and sagittal (B) conventional MR images and diffusion-weighted MR image (C). (D)–(F): preoperative axial (D) and sagittal (E) conventional MR images and diffusion-weighted MR image (F). The red circles in (B)–(F) indicate the largest pretreatment and preoperative lesion as measured in different planes and using different MRI techniques.

Clinical response was evaluated using the RECISR criteria for solid tumors (version 1.1).9 Complete response (CR) was defined as complete disappearance of all lesions; partial response (PR) was defined as at least a 30% decrease in the sum of the largest diameter (LD) of the targeted lesions; stable disease (SD) was defined as neither shrinkage that qualified as PR nor sufficient increase that qualified as progressive disease (PD); and PD was defined as at least a 20% increase in the sum of the LD of the target lesions. The overall response was defined as CR plus PR. The clinical response was evaluated based on imaging only. The images were evaluated by two experienced radiologists aware of patient diagnosis and treatment but not the results of other imaging modalities. Upon discrepancy between the two readings, a third, independent experienced radiologist served as the final arbitrator. Representative MRI and DWI of a 55-year-old patient are shown in Figure 1.

### SCC-ag Assay

Fasting serum samples were collected in duplicate at the initial visit and upon the completion of NACT. SCC-ag level was measured using an IMx SCC-ag microparticle enzyme immunoassay kit (Abbott Laboratories, Abbott Park, IL).

### Pathological Assessment

All specimens removed by surgery were submitted for pathological analysis that included macroscopic measurement of the lesion size and microscopic determination of the lesion boundary based on frozen tissue.

### Statistical Analysis

Categorical data are described as frequency counts and percentages, and the quantitative data were presented as mean ± standard deviation (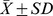). Comparisons between the clinopathological characteristics, e.g., FIGO staging, lymph node metastasis and differentiation in responsive and non-responsive patients, were performed using *Pearson*'s chi-square test. Bland-Altman analysis was used to assess the agreement between MRI examinations and pathological findings and the Bland-Altman plot was used to visualize this agreement [21]–[23]. Univariate and multivariate logistic regression analyses were conducted to determine whether SCC-ag level was an independent factor in the evaluation of the NACT response Random forest (RF) analysis [24]–[27] was carried out in the validation cohort to verify the predictive performance and compare the evaluation accuracy of MRI findings in combination with SCC-ag vs. MRI results or SCC level alone. Sensitivity (Se), specificity (Sp), positive predictive value (PPV), negative predictive value (NPV), and the area under receiver operating curve (AUC) were calculated using pathological results as reference standard.

All statistic analyses were carried using SAS version 9.1.3 (SAS, Cary, NC), with the exception of random forest analysis (R version 2.12).

## Results

### Demographic and Clinical Characteristics

**Figure 2:**
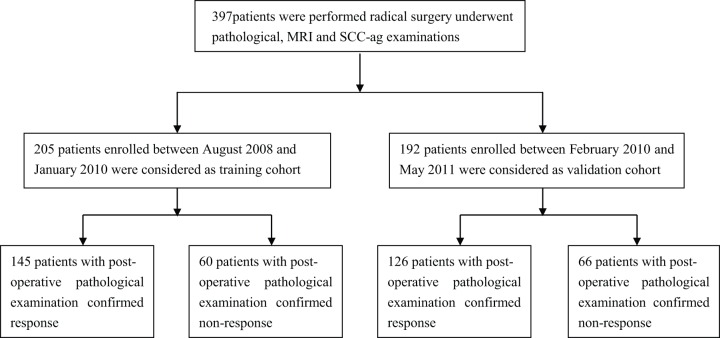
Number of patient enrollment.

Between August 2008 and October 2010, 446 subjects were enrolled. Among these, 397 patients received NACT followed by radical surgery (Figure 2). The demographic and clinical data were summarized in Table 1. The baseline characteristics were comparable across the datasets, with the exception of FIGO stage in the validation cohort. Although the training cohort had many more lymph node positive metastases, there was no significantly statistical difference (*p* = 0.0515).

**Table 1:** Demographics and Clinical Characteristics of Eligible Patients in Prospective Cohort.

Characteristics	Training cohort (n = 205)			Validation cohort (n = 192)		
	Non-response (n = 60)	Response (n = 145)	*p* value	Non-response (n = 66)	Response (n = 126)	*p* value
Age						
Mean±SD	47.65±8.39	49.1±8.42	0.2615	46.85±8.45	46.28±9.39	0.6870
Min-max	32–73	28–68		30–71	25–72	
Menses						
Non-menopause	25(41.67)	64(44.14)	0.7453	24(36.36)	41(32.54)	0.5949
menopause	35(58.33)	81(55.86)		42(63.64)	85(67.46)	
FIGO Stage						
IB2	16(26.67)	24(16.55)	0.0719	23(34.85)	22(17.46)	0.0389
IIA	12(20.00)	47(32.41)		18(27.69)	44(34.92)	
IIB	32(53.33)	74(51.03)		25(38.46)	60(47.62)	
Lymph node metastasis						
Negative	11(18.33)	46(31.72)	0.0515	35(53.03)	59(46.83)	0.4140
Positive	49(81.67)	99(68.28)		31(46.97)	67(53.17)	
Differentiation						
Well	9(15.00)	22(15.28)	0.9895	9(13.64)	32(25.4)	0.1668
Moderate	29(48.33)	68(47.22)		34(51.52)	57(45.24)	
Poor	22(36.67)	55(37.50)		23(34.85)	37(29.37)	

The values in the parenthese represented the percentage frequency;

### Agreement between MRI data and the Postsurgical Pathological Findings

**Figure 3:**
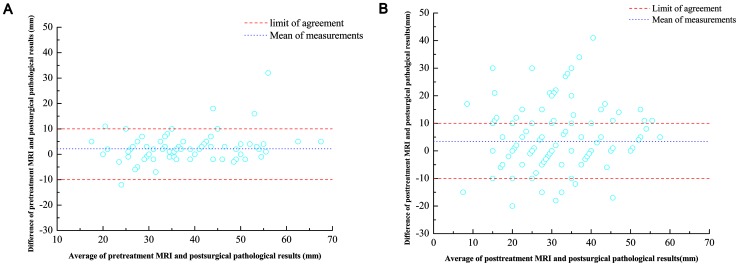
Agreement of tumor sizes, as measured by MRI versus postsurgical pathology. (A) Bland-Altman plot of tumor size measured by pretreatment MRI examination and postsurgical pathological results; 95% plots are within the limit of agreement (0±10 mm), indicating a good agreement between pretreatment MRI results and postsurgical pathological measurement. (B) Bland-Altman plot of tumor size measured by posttreatment MRI and postsurgical pathology; almost 40% plots are out of the limit of agreement (0±10 mm), which indicates a poor agreement between posttreatment MRI and postsurgical pathology, i.e posttreatment MRI results may not be in place of postsurgical pathological measurement.

The Bland-Altman plot was used to visualize the agreement between pretreatment or posttreatment MRI and the postsurgical pathological finding. In the Bland-Altman plot, the limit of agreement was the acceptably maximal difference of MRI and postsurgical pathological results, known from the clinical point of view, approximately 10 mm. The Bland-Altman analysis showed that the tumor size measured by pretreatment MRI and postsurgical pathology exhibited good agreement (Figure 3A) because 95% plots lies in the limits, whereas approximately 40% of the plots were out of the limit of agreement (Figure 3B), indicating a poor agreement in tumor size measured by posttreatment MRI and postsurgical pathological examinations results.

### SCC–ag Level and the Response to NACT

As mentioned above (see Introduction), SCC-ag is closely related to the extent of the disease as well as the response to treatment and can be used to predict the survival and tumor recurrence during the follow-up period. However, to our knowledge, few studies were performed to investigate the role of SCC-ag in the evaluation performance to chemotherapy response. Therefore, we tried to assess whether serum SCC-ag level measured before and after NACT can be a parameter for evaluation of tumor response. In this study, the 30, 50, and 70% decreases in SCC-ag levels were chosen as cutoffs to categorize the continuous pre- and post-treatment SCC-ag levels and find out the correlation between SCC-ag level and chemotherapy response in a more clear way. Univariate and multivariate logistic regression analyses of the chemotherapy response evaluation based on the MRI findings or SCC-ag levels alone were shown in Table 2. The mean and median of pre-treatment and post-treatment tumor sizes as well as range with the percent of SCC-ag decrease were presented in Table 3. Both MRI findings and SCC-ag level are independent factors for response to chemotherapy and the percent of MRI examination and SCC-ag level decrease positively correlated with chemotherapy response. From the multivariate logistic regression, we found that the percent of MRI above 30% increase the likelihood of chemotherapy response for patients by 10.28 times compared with 30% or less. The percent of SCC-ag decrease after chemotherapy exceeding 30, 50, 70% increase the possibility of chemotherapy response by 3.62, 31.70, 75.26 times compared with decrease percent below 30%, respectively.

**Table 2:** Univariate and Multivariate Logistic Analysis of SCC-ag Level and Response to Neoadjuvant Chemotherapy in a Prospective Cohort.

Variable	Univariate Analysis			Multivarate AnalysisAnalysiskuai		
	OR	95% CI	*p* value	OR	95% CI	*p* value
ΔMRI*						
<0.30	1.00	–	–	–	–	–
≥0.30	13.30	6.40–27.63	<0.0001	10.28	3.86–27.37	<.0001
ΔSCC-ag+						
<0.30	1.00	–	–	–	–	–
[0.30, 0.50)	4.63	1.53–13.98	0.0158	3.62	1.01–2.93	0.0210
[0.50, 0.70)	30.06	10.35–87.36	0.0048	31.70	9.28–108.25	0.0025
≥0.70	112.54	28.18–449.40	<.0001	75.26	17.00–333.16	<.0001

* ΔMRI indicated the decrease percentage in tumor size before and after NACT with MRI.

+ ΔSCC-ag indicated the decrease percentage in SCC-ag level before and after NACT.

**Table 3:** Tumor sizes before and after neoadjuvant chemotherapy with the percent of SCC-ag decease.

Group	Percent of SCC- ag decrease after NACT	Pretreatment		Posttreatment	
		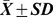	Median(range)	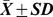	Median(range)
Training cohort (n = 205)	<0.30	42.93±13.77	44(20∼75)	35.78±14.95	37(0∼61)
	[0.30, 0.50)	43.13±12.84	40(24∼73)	30.25±14.75	28(0∼61)
	[0.50, 0.70)	45.85±10.33	44.5(25∼68)	26.92±14.36	28.5(0∼51)
	≥0.70	47.49±12.07	47(28∼80)	24.01±13.01	23.5(0∼67)
Validation cohort (n = 192)	<0.30	42.68±12.05	40(24∼68)	36.51±16.79	40(0∼65)
	[0.30, 0.50)	41.00±10.53	40(24∼60)	28.43±14.08	25(0∼54)
	[0.50, 0.70)	44.56±11.05	44(20∼68)	27.47±12.82	25(0∼50)
	≥0.70	48.89±11.41	50(24∼80)	24.02±13.56	22(0∼67)

### Validation

**Figure 4:**
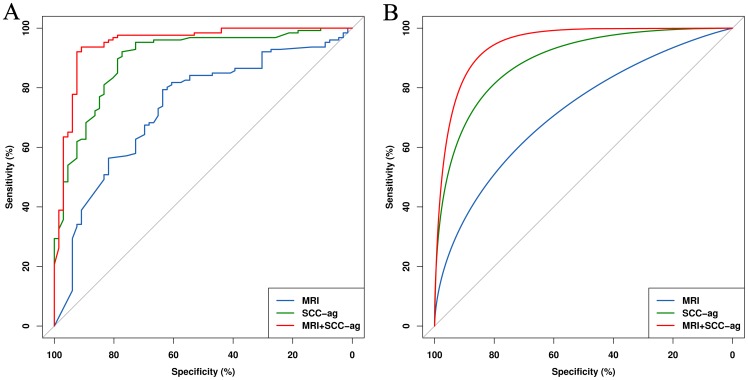
The empirical (A) and smooth (B) AUCs in the validation cohort. MRI in combination with serum SCC-ag vs. MRI or SCC-ag alone, respectively.

Analysis of the data from the validation cohort using a random forest model demonstrated that chemotherapy response could be more accurately predicted by SCC-ag than by MRI in Table 4 (Se: 0.944 vs. 0.794; Sp: 0.727 vs. 0.636; PPV: 0.869 vs. 0.806; NPV: 0.873 vs. 0.618; AUC: 0.898 vs. 0.734). Combining SCC-ag with MRI was more powerful than MRI alone (Se: 0.952 vs. 0.794; Sp: 0.833 vs. 0.636; PPV: 0.916 vs. 0.806; NPV: 0.902 vs. 0.618; AUC: 0.950 vs. 0.734). Figure 4 presented the empirical and smooth AUCs for the external validation cohort with MRI plus SCC-ag, MRI and SCC-ag alone.

**Table 4:** The Accuracy Estimation of NACT Response in an External Validation Cohort.

Evaluation indicator	ΔMRI		ΔSCC		MRI plus SCC	
	Estimator	95%CI	Estimator	95%CI	Estimator	95%CI
Sensitivity	0.80	0.72–0.87	0.94	0.90–0.98	0.95	0.91–0.98
Specificity	0.64	0.52–0.74	0.73	0.62–0.83	0.83	0.74–0.92
PPV	0.81	0.73–0.87	0.87	0.81–0.92	0.92	0.86–0.96
NPV	0.62	0.50–0.74	0.87	0.78–0.95	0.90	0.82–0.97
AUC	0.73	0.66–0.81	0.90	0.85–0.95	0.95	0.91–0.99

Abbreviation: ΔMRI: the decrease percentage in tumor size before and after NACT with MRI; ΔΔSCC-ag: the decrease percentage in SCC-ag level before and after NACT; PPV: positive predictive value; NPV: negative predictive value; AUC: area under the ROC curve;

The confidence interval was estimated based on exact test of binominal distribution.

## Discussion

The current study revealed good agreement of post-surgical pathological findings with MRI findings prior to, but not after NACT. Possible factors that contributed to the poor agreement after chemotherapy include necrosis, granuloma formation, hyaline degeneration, tiny lesions and inflammation in the target lesions after the treatment. Diffusion-weighted MRI (DW-MRI) could improve the accuracy, but only to limited degree [28], [29].

Serum SCC-ag, which is one of the most common biomarkers of cervical cancer, has been used to monitor the disease recurrence. Few studies, however, have investigated the role of the SCC-ag levels in the evaluation of NACT response in SCC patients. Hashimoto et al investigated the role of SCC-ag as a biomarker of chemotherapy response in patients with metastatic cervical cancer and reported that the patients with reduction in serum SCC-ag levels may be responsive to chemotherapy [30] and Kim et al demonstrate that there is a linear correlation between percent decrease in SCC-ag and that of tumor volume in cervical cancer patients [31], which are consistent with our results. Besides, we also demonstrates that the accuracy of the percent of SCC-ag decrease alone in the chemotherapy response evaluation is better than that of MRI alone, and SCC-ag level together with MRI examination is more powerful than MRI alone. Additionally, Reesink-Peter et al. recently reported that in the early-stage cervical cancer, serum SCC-ag level is more refined preoperative estimation of the likelihood for adjuvant raditotherapy [32]. SCC level may be useful in the evaluation of primary and recurrent SCC of cervix to radiation and chemotherapy [15], Ohara et al found that postradiotherapy SCC-ag can be used to predict tumor reccurence and not useful for evaluation radiotherapy response [33]. In this present study, we did not mention the relationship between radiotherapy response or metastasis and decrease percent of SCC-ag level, which needs to be further studied.

The ADC values from the DW-MRI data at each assessment (Table 5) did not differ between non-responsive and responsive patients, suggesting the limited value of DW-MRI.

**Table 5:** Apparent diffusion coefficient (ADC) values in the response and non-response patients before and after chemotherapy treatment (mm^2^⋅s).

	*N*	Pre-treatment	Post-treatment
Response	271	0.92±0.22×10^−3^	1.03±0.16×10^−3^ *
Non-response	126	0.93±0.18×10^−3^	1.01±0.24×10^−3^ *

* compare with pre-treatment ADC value, *p*>0.05;

In theory, the findings from the current study could be reasonably extrapolated to all malignant tumors that originate from squamous tissue. Such a speculation, however, requires extensive studies in the future.

In conclusion, our study indicates that serum SCC-ag level is a sensitive and reliable measure to evaluate cervical cancer response to chemotherapy. Using SCC-ag in combination with MRI findings further improves the predictive power. Chemotherapy response evaluation may benefit from this serum biomarker and help the oncologists to make optimal decision for cervical cancer patients.

## References

[1] Benedetti-Panici P, Maneschi F, Cutillo G, Greqqi S, Salerno MG, et al (1996) Modified type IV-V radical hysterectomy with systematic pelvic and aortic lymphadenectomy in the treatment of patients with stage III cervical carcinoma. Feasibility, technique, and clinical results. Cancer 78: 2359–2365. 8941007 10.1002/(sici)1097-0142(19961201)78:11<2359::aid-cncr14>3.0.co;2-#

[2] Iwasaka T, Fukuda K, Hara K, Yokoyama M, Nakao Y, et al (1998) Neoadjuvant chemotherapy with mitomycin C, etoposide, and cisplatin for adenocarcinoma of the cervix. Gynecol Oncol 70: 236–240. 9740697 10.1006/gyno.1998.5079

[3] deSouza NM, Soutter WP, Rustin G, Mahon MM, Jones B, et al (2004) Use of neoadjuvant chemotherapy prior to radical hysterectomy in cervical cancer: monitoring tumor shrinkage and molecular profile on magnetic resonance and assessment of 3-year outcome. Br J Cancer 90: 2326–2331. 15162152 10.1038/sj.bjc.6601870 PMC2409522

[4] Yin M, Zhao F, Lou G, Zhang H, Sun M, et al (2011) The long-term efficacy of neoadjuvant chemotherapy followed by radical hysterectomy compared with radical surgery alone or concurrent chemoradiotherapy on locally advanced stage cervical cancer. Int J Gynecol Cancer 21: 92–99. 21330834 10.1111/IGC.0b013e3181fe8b6e

[5] Panici PB, Scambia G, Baiocchi G, Greggi S, Ragusa G, et al (1991) Neoadjuvant chemotherapy and radical surgery in locally advanced cervical cancer. Prognostic factors for response and survival. Cancer 67: 372–379. 1702348 10.1002/1097-0142(19910115)67:2<372::aid-cncr2820670210>3.0.co;2-5

[6] Park DC, Kim JH, Lew YO, Kim DH, Namkoong SE (2004) Phase II trial of neoadjuvant paclitaxel and cisplatin in uterine cervical cancer. Gynecol Oncol 92: 59–63. 14751139 10.1016/j.ygyno.2003.09.015

[7] Micheletti E, La Face B, Bianchi E, Caqna E, Apostoli P, et al (1997) Continuous infusion of carboplatin during conventional radiotherapy treatment in advanced squamous carcinoma of the cervix uteri IIB-IIIB (UICC). A phase I/II and pharmacokinetic study. Am J Clin Oncol 20: 613–620. 9391552 10.1097/00000421-199712000-00017

[8] Peters WA, Liu PY, Barrett RJ, Stock RJ, Monk BJ, et al (2000) Concurrent chemotherapy and pelvic radiation therapy compared with pelvic radiation therapy alone as adjuvant therapy after radical surgery in high-risk early-stage cancer of the cervix. J Clin Oncol 18: 1606–1613. 10764420 10.1200/JCO.2000.18.8.1606

[9] Eisenhauer EA, Therasse P, Bogaerts J, Schwartz LH, Sargent D, et al (2009) New response evaluation criteria in solid tumours: revised RECIST guideline (version 1.1). Eur J Cancer 45: 228–247. 19097774 10.1016/j.ejca.2008.10.026

[10] Vincens E, Balleyquier C, Rey A, Uzan C, Zareski E, et al (2008) Accuracy of Magnetic Resonance Imaging in Predicting Residual Disease in Patients Treated for Stage IB2/II Cervical Carcinoma With Chemoradiation Therapy. Cancer 113: 2158–2165. 18780318 10.1002/cncr.23817

[11] Dimitrakopoulou-Strauss A, Strauss LG, Eqerer G, Vasamiliette J, Mechtersheimer G, et al (2010) Impact of dynamic 18F-FDG PET on the early prediction of therapy outcome in patients with high-risk soft-tissue sarcomas after neoadjuvant chemotherapy: a feasibility study. J Nucl Med 51(4): 551–558. 20351350 10.2967/jnumed.109.070862

[12] Kato H, Torigoe T (1977) Radioimmunoassay for tumor antigen of human cervical squamous cell carcinoma. Cancer 40: 1621–1628. 332328 10.1002/1097-0142(197710)40:4<1621::aid-cncr2820400435>3.0.co;2-i

[13] Kato H, Morioka H, Tsutsui H, Aramaki S, Torigoe T (1982) Value of tumorantigen (TA-4) of squamous cell carcinoma antigen in predicting the extent of cervical cancer. Cancer 50: 1294 –1296. 10.1002/1097-0142(19821001)50:7<1294::aid-cncr2820500712>3.0.co;2-k 7104971

[14] Scambia G, Benedetti Panici P, Foti E, Amoroso M, Salerno G, et al (1994) Squamous cell carcinoma antigen: Prognostic significance and role in the monitoring of neoadjuvant chemotherapy response in cervical cancer. J Clin Oncol 12: 2309–2316. 7964945 10.1200/JCO.1994.12.11.2309

[15] Yazigi R, Munoz AK, Richardson B, Risser R (1991) Correlation of squamous cell carcinoma antigen levels and treatment response in cervical cancer. Gynecol Oncol 41: 135–138. 2050303 10.1016/0090-8258(91)90272-7

[16] Ngan HYS, Chan SYW, Wong LC, Choy DTK, Ma HK (1990) Serum squamous cell carcinoma antigen in the monitoring of radiotherapy treatment response in carcinoma of the cervix. Gynecol Oncol 37: 260 –263. 10.1016/0090-8258(90)90344-k 2344971

[17] Strauss HG, Laban C, Lautenschlager C, Buchmann J, Schmann J, et al (2002) SCC antigen in the serum as an independent prognostic factor in operable squamous cell carcinoma of the cervix. Eur J Cancer 38: 1987–1991. 12376202 10.1016/s0959-8049(02)00159-4

[18] Bae SN, Namkoong SE, Jung JK, Kim CJ, Park JS, et al (1997) Prognostic significance of pre-treatment squamous cell carcinoma antigen and carcinoembryonic antigen in squamous cell carcinoma of the uterine cervix. Gynecol Oncol 64: 418–424. 9062143 10.1006/gyno.1996.4589

[19] Massuger LF, Koper NP, Thomas CM, Dom KF, Schijf CP (1997) Improvement of clinical staging in cervical cancer with serum squamous cell carcinoma antigen and CA 125 determinations. Gynecol Oncol 64: 473–476. 9062153 10.1006/gyno.1996.4581

[20] Chou CY, Wang ST, Kuo HC, Tzeng CC, Yao BL (1994) Serum level of squamous cell carcinoma antigen and tumor size are useful to identify preoperatively patients at high risk of cervical cancer. Cancer 74: 2497–2501. 7923006 10.1002/1097-0142(19941101)74:9<2497::aid-cncr2820740917>3.0.co;2-l

[21] Bland JM, Altman DG (1986) Statistical methods for assessing agreement between two methods of clinical measurement. Lancet 1: 307–310. 2868172

[22] Hüttmann EM, Greulich T, Hattesohl A, Schmid S, Noeske S, et al (2011) Comparison of two devices and two breathing patterns for exhaled breath condensate sampling. PLoS One 6: e27467. 22087323 10.1371/journal.pone.0027467 PMC3210176

[23] Er F, Ederer S, Nia AM, Caglayan E, Dahlem KM, et al (2011) Accuracy of Doppler-echocardiographic mean pulmonary artery pressure for diagnosis of pulmonary hypertension. PLoS One 5: e15670. 10.1371/journal.pone.0015670 PMC3003692 21179417

[24] Breiman L (2001) Random Forest. Machine Learning 45: 5–32.

[25] Khondoker MR, Bachmann TT, Mewissen M, Dickinson P, Dobrzelecki B, et al (2010) Multi-factorial analysis of class prediction error: estimating optimal number of biomarkers for various classification rules. J Bioinform Comput Biol 8: 945–965. 21121020 10.1142/s0219720010005063

[26] Borgia JA, Basu S, Faber LP, Kim AW, Coon JS, et al (2009) Establishment of a multi-analyte serum biomarker panel to identify lymph node metastases in non-small cell lung cancer. J Thorac Oncol 4: 338–347. 19190517 10.1097/JTO.0b013e3181982abf

[27] Hamby SE, Hirst JD (2008) Prediction of glycosylation sites using random forests. BMC Bioinformatics 9: 500. 19038042 10.1186/1471-2105-9-500 PMC2651179

[28] Kwee TC, Takahara T, Ochiai R, Nievelstein RA, Luijten PR (2008) Diffusion-weighted whole-body imaging with background body signal suppression (DWIBS): features and potential applications in oncology. Eur Radiol 18: 1937–1952. 18446344 10.1007/s00330-008-0968-z PMC2516183

[29] Koh DM, Cook GJ, Husband JE (2003) New horizons in oncologic imaging. N Engl J Med 348: 2487–2488. 12815132 10.1056/NEJMp030048

[30] Hashimoto K, Yonemori K, Katsumata N, Hirakawa A, Hirata T, et al (2011) Use of squamous cell carcinoma antigen as a biomarker of chemotherapy response in patients with metastatic cervical carcinoma. Eur J Obstet Gynecol Reprod Biol 159(2): 394–398. 21831511 10.1016/j.ejogrb.2011.07.001

[31] Kim BG, Kim JH, Park SY, Lee JH, Lee ED, et al (1996) Relationship between squamous cell carcinoma antigen levels and tumor volumes in patients with cervical carcinomas undergoing neoadjuvant chemotherapy. Gynecol Oncol 63: 105–113. 8898178 10.1006/gyno.1996.0287

[32] Reesink-Peters N, van der Velden J, Ten Hoor KA, Boezen HM, de Vries EG, et al (2005) Preoperative Serum Squamous Cell Carcinoma Antigen Levels in Clinical Decision Making for Patients With Early-Stage Cervical Cancer. J Clin Oncol 23: 1455–1462. 15735121 10.1200/JCO.2005.02.123

[33] Ohara K, Tanaka Y, Tsunoda H, Nishida M, Sugahara S, et al (2002) Assessment of Cervical Cancer Radioresponse by Serum Squamous Cell Carcinoma Antigen and Magnetic Resonance Imaging. Obstet Gynecol 100: 781–787. 12383549 10.1016/s0029-7844(02)02204-4

